# Analysis of Career Stage, Gender, and Personality and Workplace Violence in a 20-Year Nationwide Cohort of Physicians in Norway

**DOI:** 10.1001/jamanetworkopen.2021.14749

**Published:** 2021-06-28

**Authors:** Sara Tellefsen Nøland, Hildegunn Taipale, Javed Iqbal Mahmood, Reidar Tyssen

**Affiliations:** 1 Faculty of Medicine, University of Oslo, Oslo, Norway; 2 Institute of Basic Medical Sciences, Department of Behavioural Medicine, Faculty of Medicine, University of Oslo, Oslo, Norway; 3 Emergency Addiction Services Consulting Team, Oslo University Hospital, Oslo, Norway

## Abstract

**Importance:**

Workplace violence (WPV) is a worldwide problem in health services. Several studies have pointed to organizational factors, such as working in psychiatry and work stress. However, there is a lack of long-term longitudinal cohort studies with respect to trends during the career and individual factors among physicians.

**Objective:**

To investigate WPV trends during Norwegian physicians’ careers and assess individual and work-related factors associated with WPV in a long-term longitudinal study.

**Design, Setting, and Participants:**

This cohort study involved 2 nationwide medical student cohorts who graduated 6 years apart and were surveyed at graduation (T1: 1993-1994 and 1999) and 4 years later (T2), 10 years later (T3), 15 years later (T4), and 20 years after graduation (T5). Generalized estimated equations were used. Statistical analysis was performed from January to September 2020.

**Exposures:**

Medical career during 20 years in Norway.

**Main Outcomes and Measures:**

WPV was measured as threats or acts of violence from a patient or visitor experienced at least twice, at each of the stages after leaving medical school. Individual factors were obtained at T1 and work-related factors at T2 through T5. We analyzed WPV by repeated measures.

**Results:**

At T1, a total of 893 participants (with a mean [SD] age of 28 (2.83) years; 499 [56%] women) responded to the questionnaire. The prevalence of multiple threats of violence was 20.3% (156 of 769) at T2, 17.1% (118 of 691) at T3, 11.2% (66 of 588) at T4, and 8.6% (46 of 536) at T5; and the prevalence of multiple acts of violence was 4.3% (33 of 763) at T2, 5.2% (36 of 687) at T3, 3.1% (18 of 584) at T4, and 2.2% (12 of 532) at T5. There was a decline from T2 to T5 of both multiple threats (β = −1.06; 95% CI, −1.31 to −0.09; *P* < .001) and acts of violence (β = −1.13; 95% CI, −1.73 to −0.53; *P* < .001). In adjusted analysis, factors associated with multiple threats of violence were male gender (odds ratio [OR], 2.76; 95% CI, 1.73 to 4.40; *P* < .001), vulnerability trait (neuroticism) (OR, 0.90; 95% CI, 0.82 to 0.99; *P* = .03), young physician cohort (OR, 1.63; 95% CI, 1.04 to 2.58; *P* = .04), and working in psychiatry (OR, 7.50; 95% CI, 4.42 to 12.71; *P* < .001). Factors associated with multiple acts of violence in adjusted analysis were male gender (OR, 3.37; 95% CI, 1.45 to 7.84; *P* = .005), young physician cohort (OR, 6.08; 95% CI, 1.68 to 21.97; *P* = .006), and working in psychiatry (OR, 12.34; 95% CI, 5.40 to 28.23; *P* < .001). There were no interactions with gender or cohort in the significant associated factors.

**Conclusions and Relevance:**

Higher rates of multiple threats and acts of violence were observed during early medical careers, among male physicians, and in psychiatry. Low levels of the vulnerability trait (neuroticism) were associated with the experience of multiple threats. There was an association between the young physician cohort and WPV. Preventive efforts should include early-career and male physicians, with additional emphasis on personality.

## Key Points

**Question:**

What are the trends of workplace violence during physicians’ careers, and what are the individual and work-related factors of experiencing workplace violence in a long-term follow-up?

**Findings:**

In this 20-year longitudinal study of 2 cohorts including 893 Norwegian physicians surveyed 6 years apart, the prevalence of multiple threats and acts of violence declined throughout physicians’ careers. Both threats and acts of violence were significantly associated with male gender, young physician cohort, and psychiatry; threats of violence were also associated with low levels of the vulnerability trait.

**Meaning:**

These findings suggest that efforts to prevent workplace violence should include early-career and male physicians and may benefit from focusing on physician personality traits.

## Teaser

This cohort study examines the incidence of violence and threats of violence against physicians in Norway during a 20-year period.

## Introduction

There is a high prevalence of both threats and acts of violence against health care professionals globally, which is often classified as workplace violence (WPV).^1,2^ Previous studies have reported severe individual consequences and high societal costs following acts and threats of violence.^1,2,3,4,5,6,7^ The incidence of reported physical violence against health care professionals around the world ranges from 4% to 37%.^2^ In Norway, as many as one-half of physicians have experienced threats of violence at least once and one-quarter have experienced an act of violence from a patient.^8^ However, there is currently a lack of longitudinal studies that describe the trends in WPV during a medical career and work-related factors associated with WPV after controlling for individual factors. These studies can identify factors that could inform interventions to increase safety among physicians and other health care professionals.

By measuring WPV as multiple acts or threats of violence, we may identify more enduring individual and structural associated factors, and with higher reliability than assessing single incidents. To our knowledge, there is a lack of information about these factors from long-term longitudinal studies. Among the medical specialties, physicians in psychiatry are at the highest risk of experiencing WPV, followed by those working in emergency departments and general practice.^9,10,11^ This applies to the perpetrator being a patient or visitor, which may be classified as type II WPV.^1^ In the US, the rate of violence in psychiatric departments is 69 times the national rate of violence in the workplace.^1^ In Norwegian psychiatric departments, 46% of physicians have experienced acts of violence.^8^

Studies also point to possible risk factors such as understaffing,^12^ high patient-contact time,^1^ and excessive workload.^13^ A previous study observed a reduction in threats and acts of violence among Norwegian physicians in psychiatry during the years 1993 to 2004,^8^ which may have been because of an increased focus on prevention of violence in local hospital departments.

Whether there is a gender difference in WPV or not is unclear. There is some evidence of both a higher exposure among men^14,15,16,17^ and no gender difference.^18^ The possible gender difference is often not addressed in studies of health care workers and studies can be gender-balanced poorly.^2^

The present study used new and original data from the 25-year follow-up in 2019 of the Longitudinal Study of Norwegian Medical Students and Doctors (NORDOC), which allowed us to follow associated factors of experiencing multiple acts or threats of violence at 4 stages of physicians’ medical career at intervals of approximately 5 years. There may be differences in proneness to WPV among individuals during different stages of their medical career. Cross-sectional have studies indicated that early-career health professionals experience violent incidents from patients more often^19,20^; however, only a few Western studies have investigated this question, where the impact of career stage is uncertain.^8^ Thus, we lack suitable longitudinal studies on this issue. NORDOC followed 2 nationwide cohorts who graduated 6 years apart in 1993 to 1994 and 1999, which allowed us to study any associations between cohort and WPV. We would expect a reduction in WPV during more recent years, as is shown in a past Norwegian cross-sectional sample.^8^

This study examined the following questions: What is the course or trends of WPV during 20 years of a medical career? Are there any particular work-related or individual characteristics, such as gender or personality, that are associated with WPV?

## Methods

This study was conducted according to the guidelines of the Regional Committee for Medical Research. This study was also approved by the Norwegian Centre for Research Data. The identity of the responders was kept anonymous by storing this information at the Central Bureau of Statistics, Norway. This study followed the Strengthening the Reporting of Observational Studies in Epidemiology (STROBE) reporting guideline for cohort studies.

### The NORDOC Sample

Two nationwide cohorts (ie, medical student and young physician cohorts) who graduated 6 years apart in 1993 to 1994 and 1999 were followed for 20 years after graduation. We reported a 15-year follow-up of both cohorts,^21,22^ and a 20-year follow-up of the young physician cohort previously.^23,24^ Medical students from all 4 Norwegian universities were included. Participants in the NORDOC survey were compensated with a gift card worth approximately $20.

The present data were obtained at 5 time points: T1, final year of medical school (data collected in 1993-1994 and 1999, 74% of the eligible sample [893 of 1211 medical students]); T2, 4 years after graduation (data collected in 1998 and 2003, 68% of the eligible sample [780 of 1140 physicians]); T3, 10 years after graduation (data collected in 2003 and 2008, 71% of the eligible sample [708 of 1004 physicians]); T4, 15 years after graduation (data collected in 2008 and 2014, 60% of the eligible sample [589 of 982 physicians]); and T5, 20 years after graduation (data collected in 2014 and 2019, 57% of the eligible sample [558 of 972 physicians]). When reviewing the data file, 1052 participants had responded at least once.

We collected information about race/ethnicity in NORDOC 2014 owing to sampling of DNA. However, race/ethnicity have not been used in any of the analyses thus far, mainly because the sample included a large majority of White individuals (96%).

### Measures

#### Dependent Variables

Threats and acts of violence were measured repeatedly from T2 through T5. At T2 and T3, participants were asked: “Have you ever experienced acts or threats of violence from a patient or a visitor?” At T4 and T5, participants were asked: “Have you experienced acts or threats of violence from a patient or a visitor since last time?” The latter referred to a period of about 5 years. For both questions, the response choices were 1 through 4: (1) no, (2) yes, once, (3) yes, twice, and (4) yes, more than twice. To measure and analyze multiple threats or acts of violence (occurred twice or more), we recorded and dichotomized the variable by setting responses 3 or 4 equal to 1, and responses 1 or 2 were equal to 0. The Figure illustrates the sample and study design details.

**Figure.**
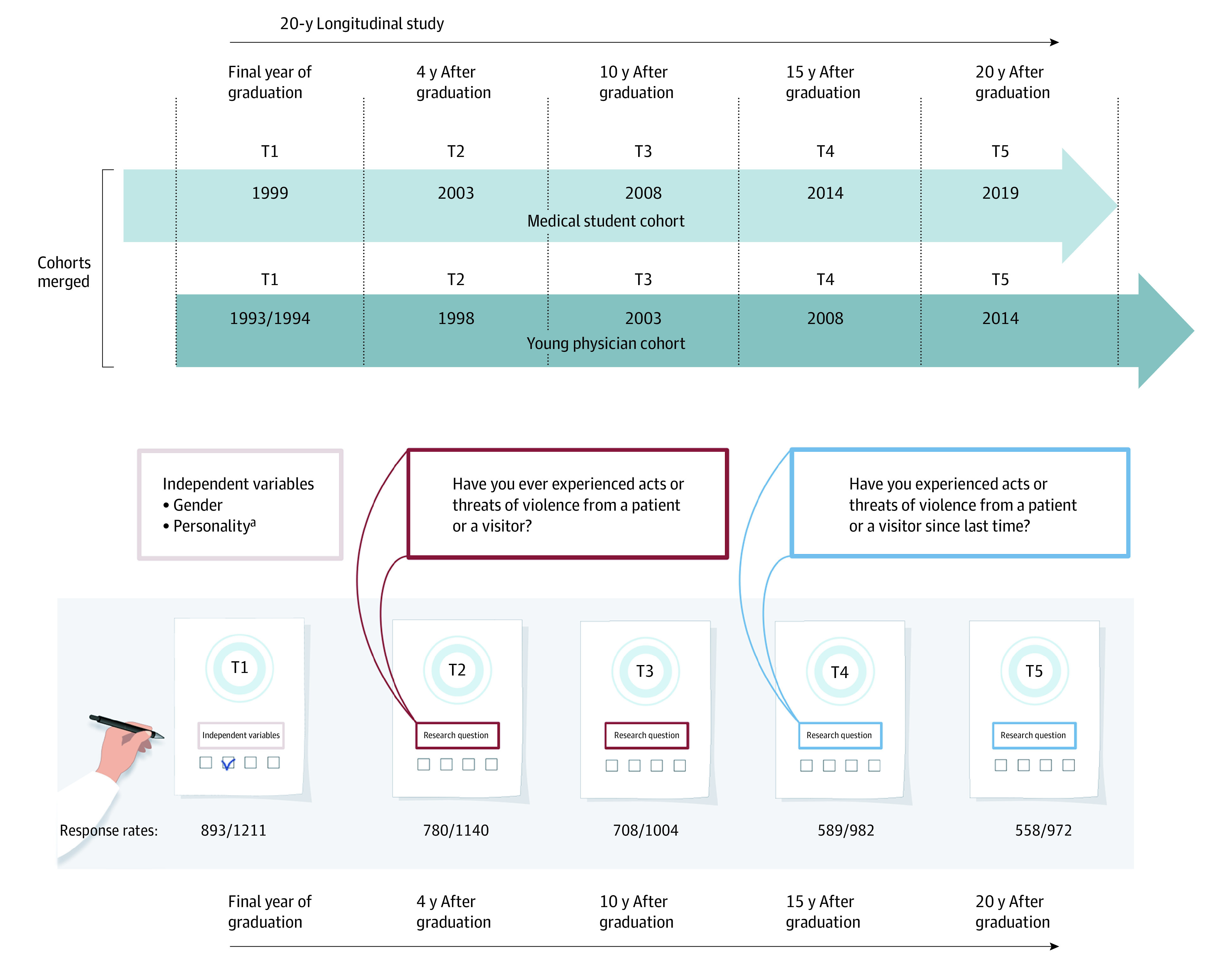
Overview of the Survey Through 20 Years ^a^Gathered at baseline in 1993 in the medical student cohort during their first year at medical school, and gathered at T1 and 1 year later in the young physician cohort.

#### Independent Variables

Age was measured as a continuous variable. Gender was coded as 0 = woman and 1 = man. The cohort was coded as 0 = medical students and 1 = young physicians.

#### Personality

Personality traits were measured at baseline using the 36-item version of the Torgersen basic character inventory (BCI-36), which assesses 4 personality trait dimensions: vulnerability, intensity, control, and reality weakness. The first 3 dimensions resemble the Eysenck Giant Three^25^ (ie, neuroticism, extroversion, and conscientiousness). Reality weakness is a deviant dimension involving perceptions and ideation at the borderline between reality and fantasy, and it measures traits linked to severe personality disorders.^26^

Each dimension is based on 9 questions, each of which has a dichotomous response (agree or do not agree). The total score can range from 0 (low) to 9 (high). The sum score of each personality trait dimension was used in the analysis. Data from personality variables in the medical student cohort were gathered at baseline in 1993 during their first year at medical school, whereas these data were gathered at T1 and 1 year later in the young doctor cohort.^27,28^ The BCI-36 was validated in previous Norwegian studies.^21,29,30,31^

#### Mental Distress

Mental distress during the past 2 weeks was measured at T2, T3, and T4 using the 5-item Hopkins Symptom Checklist (SCL-5), a shorter version of the SCL-25.^32,33^ The SCL-5 indicated how much a person had been bothered by each of the 5 specific symptoms during the past 2 weeks. Responses were answered on a 5-point Likert scale (1 = not at all to 5 = very much). The mean item score was used to assess the level of mental distress. This scale has been used in general population and Norwegian physician studies.^32,34^

#### Clinical Specialty

The questionnaires all had 9 specialty options, but in this analysis, only 2 categories were used: psychiatry and general practitioners at T4. In Norway, general practitioners are on-call staff in the emergency primary care departments.

#### Job Stress

Perceived job stress was measured at T2, T3, T4, and T5 using a 32-item scale, which was a modified version of the Cooper Job Stress Questionnaire for general practitioners.^35^ It was later modified and validated among Norwegian physicians.^27,36^ Each item was measured on a 5-point Likert scale (1 = no stress to 5 = much stress). A factor analysis of data at T2^36^ identified 4 dimensions: emotional pressure (8 items), time pressure (6 items), fear of complaints or criticism (7 items), and work-home interference (3 items). The mean item score of each dimension assessed the level of perceived job stress. The mean number of working hours per week was measured at T2, T3, T4, and T5 (Table 1).

**Table 1.** Overview and Description of the Sample

Independent variables	T1, final y of education (n = 893)	T2, 4 y after graduation (n = 780)	T3, 10 y after graduation (n = 708)	T4, 15 y after graduation (n = 589)	T5, 20 y after graduation (n = 558)
Age, y, [continuous], mean (SD)	28 (2.83)	NA	NA	NA	NA
Mental distress, mean (SD)	1.81 (0.79)	1.50 (0.64)	1.48 (0.63)	1.44 (0.58)	1.53 (0.73)
Gender, No. (%)^a^					
Women	499 (56)	NA	NA	NA	NA
Men	388 (43)	NA	NA	NA	NA
Personality traits, mean (SD)^b^					
Vulnerability	3.52 (2.23)	NA	NA	NA	NA
Reality weakness	1.44 (1.59)	NA	NA	NA	NA
Control	3.09 (2.07)	NA	NA	NA	NA
Intensity	5.46 (2.36)	NA	NA	NA	NA
Contextual work-related variables					
General practice %	NA	29.7^c^	23.0^c^	21.9	NA^d^
Psychiatry %	NA	8.4^c^	10.4^c^	10.4	NA^d^
Work hours per week, mean (SD)	NA	45.29 (9.91)	42.99 (8.89)	42.21 (10.96)	44.94 (10.53)
Emotional pressure, mean (SD)^e^	NA	2.07 (0.65)	1.86 (0.58)	1.79 (0.53)	1.80 (0.62)
Time pressure, mean (SD)^e^	NA	2.41 (0.69)	2.25 (0.68)	2.16 (0.67)	2.16 (0.68)
Fear of complaints and criticism, mean (SD)^e^	NA	2.11 (0.67)	1.93 (0.65)	1.80 (0.56)	1.79 (0.60)
Work-home interference, mean (SD)^e^	NA	2.46 (1.00)	2.42 (1.00)	2.33 (0.99)	2.29 (1.00)

Abbreviation: BCI-36, 36-item version of the Torgersen basic character inventory; NA, not applicable.

^a^ Six participants did not report gender data.

^b^ Personality traits were measured only at baseline using the BCI-36 (see Methods section).

^c^ Specialty percentages at T2 and T3 were not included in the analyses.

^d^ Specialty was not included at T5.

^e^ Job stress factors were measured using a modified version of the Cooper Job Stress Questionnaire (see Methods section).

### Statistical Analysis

Statistical analyses were performed using SPSS software version 20 (IBM Corp), using generalized-estimating equations (GEE) with an unstructured covariance matrix and robust estimator. GEE is a repeated-measure mixed model for categorical outcomes, which in this study was binary. The method considers dependency between the observation periods and includes all available data for each participant, such as being less affected by the missing data. We were interested in the fixed effects of the specified factors as hypothesized, and we did not include random effects in the model. First, we performed an unadjusted analysis of the associations of all independent variables (including the cohort) measured at T1 and contextual work-related variables and mental distress (measured at T2 through T4) on multiple threats and acts of violence, respectively, as dependent variables at T2 through T5. The independent variables were then entered into 2 blocks in the adjusted analyses to separate the associations of individual factors from contextual work-related factors. Reported *P* values were 2-sided and considered statistically significant at *P* < .05. Statistical analyses were performed from January to September 2020.

## Results

At T1, of 1211 medical students and young physicians who received a questionnaire, a total of 893 responded (74%). The mean (SD) age was 28 (2.83) years; 499 (56%) were women. Table 1 shows a description of the variables among the respondents.

### Prevalence of Multiple Threats of Violence

Table 2 shows the prevalence of multiple (≥2) threats of violence for the various time periods. The prevalence was 20.3% (156 of 769) at T2 (14.5% [63 of 433] for women vs 27.7% [93 of 336] for men; *P* < .001), 17.1% (118 of 691) at T3 (11.3% [45 of 399] for women vs 25.0% [73 of 292] for men; *P* < .001), 11.2% (66 of 588) at T4 (9.1% [32 of 352] for women vs 14.4% [34 of 236] for men [no significant difference]), 8.6% (46 of 536) at T5 (7.3% [23 of 316] for women vs 10.5% [23 of 220] for men [no significant difference]). There was a significant decline in the prevalence of multiple threats of violence from T2 to T5 (β = −1.06; 95% CI, −1.31 to −0.09; *P* < .001).

**Table 2.** Prevalence of Violence or Threat of Violence Experienced by Physicians

Prevalence	4 y after graduation^a^		10 y after graduation^a^		15 y after graduation^b^		20 y after graduation^b^	
	Incidents, No./total No. (%)^c^	*P* value for gender difference	Incidents, No./total No. (%)^c^	*P* value for gender difference	Incidents, No./total No. (%)^c^	*P* value for gender difference	Incidents, No./total No. (%)^c^	*P* value for gender difference
Experienced threat of violence at least once								
All	286/769 (37.2)	<.001	229/691 (33.1)	<.001	144/588 (24.4)	.56	91/536 (17.0)	.08
Male	152/336 (45.2)		126/292 (43.2)		61/236 (25.8)		45/220 (20.5)	
Female	134/433 (30.9)		103/399 (25.8)		83/352 (23.6)		46/316 (14.6)	
Experience threat of violence twice or more								
All	156/769 (20.3)	<.001	118/691 (17.1)	<.001	66/588 (11.2)	.06	46/536 (8.6)	.21
Male	93/336 (27.7)		73/292 (25.0)		34/236 (14.4)		23/220 (10.5)	
Female	63/433 (14.5)		45/399 (11.3)		32/352 (9.1)		23/316 (7.3)	
Experienced violence at least once								
All	101/763 (13.2)	.02	92/687 (13.4)	<.001	43/584 (7.4)	.15	26/532 (4.9)	.68
Male	55/335 (16.4)		55/291 (18.9)		22/235 (9.4)		12/217 (5.5)	
Female	46/428 (10.7)		37/396 (9.3)		21/349 (6.0)		14/315 (4.4)	
Experienced violence twice or more								
All	33/763 (4.3)	.03	36/687 (5.2)	.003	18/584 (3.1)	.22	12/532 (2.2)	.56
Male	21/335 (6.3)		24/291 (8.2)		10/235 (4.3)		6/217 (2.8)	
Female	12/428 (2.8)		12/396 (3.0)		8/349 (2.3)		6/315 (1.9)	

^a^ Have you ever experienced acts or threats of violence from a patient or a visitor?

^b^ Have you experienced acts or threats of violence from a patient or a visitor since last time?

^c^ Denominator shows the number of respondents to the specific question at each time point.

### Prevalence of Multiple Acts of Violence

Table 2 shows the prevalence of multiple (≥2) acts of violence for the various time periods. The prevalence was 4.3% (33 of 763) at T2 (2.8% [12 of 428] for women vs 6.3% [21 of 335] for men; *P* = .03), 5.2% [36 of 687] at T3 (3.0% [12 of 396] for women vs 8.2% [24 of 291] for men; *P* = .003), 3.1% (18 of 584) at T4 (2.3% [8 of 349] for women vs 4.3% [10 of 235] for men [no significant difference]) and 2.2% (12 of 532) at T5 (1.9% [6 of 315] for women vs 2.8% [6 of 217] for men [no significant difference]). There was a significant decline in the prevalence of multiple acts of violence from T2 to T5 (β = −1.13, 95% CI, −1.73 to −0.53; *P* < .001).

### Factors Associated With Multiple Threats of Violence Against Physicians

The unadjusted and adjusted factors associated with multiple (≥2) threats of violence against physicians are shown in Table 3. The adjusted factors were male gender (odds ratio [OR], 2.76; 95% CI, 1.73-4.40; *P* < .001), low vulnerability (OR, 0.90; 95% CI, 0.82–0.99; *P* = .03), young physician cohort (OR, 1.63; 95% CI, 1.04 to 2.58; *P* = .04) and psychiatry specialty (OR, 7.50; 95% CI, 4.42-12.71; *P* < .001). None of the significant adjusted factors displayed any interaction with gender or cohort, indicating that their association with WPV was not significantly different across gender or cohort.

**Table 3.** Factors Associated With Multiple Threats of Violence

Factors	Unadjusted		Adjusted			
			Model 1 (n = 641)^a^		Model 2 (n = 370)^b^	
	OR (95% CI)	*P* value	OR (95% CI)	*P* value	OR (95% CI)	*P* value
Block 1: individual factors						
Age	1.07 (1.02-1.13)	.007	1.05 (0.99-1.12)	.11	1.05 (0.96-1.16)	.31
Gender (male = 1)	2.11 (1.60-2.79)	<.001	2.13 (1.51-3.01)	<.001	2.76 (1.73-4.40)	<.001
Mental distress	0.98 (0.95-1.01)	.22	NA	NA	NA	NA
Vulnerability	0.91 (0.85-0.97)	.005	0.96 (0.89-1.03)	.23	0.90 (0.82-0.99)	.03
Reality weakness	0.93 (0.84-1.03)	.16	NA	NA	NA	NA
Control	0.89 (0.81-0.97)	.008	0.91 (0.82-0.99)	.03	0.88 (0.77-1.01)	.08
Intensity	1.08 (1.01-1.16)	.02	1.06 (0.98-1.14)	.15	0.99 (0.90-1.09)	.78
Cohort (student = 0; physician = 1)	1.38 (1.04-1.83)	.02	1.92 (1.36-2.72)	<.001	1.63 (1.04-2.58)	.04
Block 2: contextual work-related factors						
General practice	0.86 (0.56-1.34)	.51	NA	NA	NA	NA
Psychiatry	6.68 (4.14-10.76)	<.001	NA	NA	7.50 (4.42-12.71)	<.001
Work hours per week	1.003 (0.99-1.02)	.67	NA	NA	NA	NA
Job stress (4 factors)						
1: Emotional pressure	1.53 (1.28-1.82)	<.001	NA	NA	1.13 (0.69-1.83)	.63
2: Time pressure	1.37 (1.17-1.60)	<.001	NA	NA	1.31 (0.93-1.85)	.12
3: Fear of complaints and criticism	1.57 (1.33-1.84)	<.001	NA	NA	1.49 (0.99-2.25)	.06
4: Work-home interference	1.17 (1.04-1.32)	.007	NA	NA	0.86 (0.66-1.11)	.23

Abbreviations: NA, not applicable; OR, odds ratio.

^a^ Adjusted for significant factors in block 1: individual factors.

^b^ Adjusted for all significant factors in block 1 and block 2.

### Factors Associated With Multiple Acts of Violence Against Physicians

The unadjusted and adjusted factors associated with multiple (≥2) acts of violence against physicians are shown in Table 4. The adjusted factors were male gender (OR, 3.37; 95% CI 1.45–7.84; *P* = .005), young physician cohort (OR, 6.08; 95% CI, 1.68-21.97; *P* = .006), and psychiatry specialty (OR, 12.34; 95% CI, 5.40-28.23; *P* < .001). None of the adjusted factors displayed a significant interaction with gender or cohort.

**Table 4.** Factors Associated With Multiple Acts of Violence

Factors	Unadjusted		Adjusted			
			Model 1 (n = 717)^a^		Model 2 (n = 374)^b^	
	OR (95% CI)	*P* value	OR (95% CI)	*P* value	OR (95% CI)	*P* value
Block 1: individual factors						
Age	1.05 (0.97-1.14)	.20	NA	NA	1.15 (0.99-1.34)	.08
Gender (male = 1)	2.22 (1.34-3.68)	.002	2.02 (1.11-3.65)	.02	3.37 (1.45-7.84)	.005
Mental distress	0.99 (0.93-1.04)	.65	NA	NA	NA	NA
Vulnerability	0.92 (0.81-1.05)	.24	NA	NA	NA	NA
Reality weakness	1.10 (0.94-1.28)	.22	NA	NA	NA	NA
Control	0.77 (0.66-0.90)	.001	0.77 (0.66-0.91)	.002	0.76 (0.57-1.02)	.07
Intensity	1.07 (0.95-1.21)	.26	NA	NA	NA	NA
Cohort (student = 0, physician = 1)	1.85 (1.09-3.14)	.02	3.03 (1.54-5.95)	.001	6.08 (1.68-21.97)	.006
Block 2: contextual work-related factors						
General practice	0.53 (0.20-1.41)	.20	NA	NA	NA	NA
Psychiatry	12.1 (6.02-24.29)	<.001	NA	NA	12.34 (5.40-28.23)	<.001
Work hours per week	1.02 (0.20-1.04)	.12	NA	NA	NA	NA
Job stress (4 factors)						
1: Emotional pressure	1.55 (1.10-2.18)	.01	NA	NA	0.91 (0.40-2.06)	.82
2: Time pressure	1.49 (1.10-2.02)	.01	NA	NA	0.89 (0.35-2.24)	.80
3: Fear of complaints and criticism	1.63 (1.25-2.13)	<.001	NA	NA	1.72 (0.77-3.87)	.19
4: Work-home interference	1.37 (1.12-1.68)	.002	NA	NA	1.14 (0.74-1.77)	.55

Abbreviations: NA, not applicable; OR, odds ratio.

^a^ Adjusted for significant factors in block 1: individual factors.

^b^ Adjusted for all significant factors in block 1 and block 2.

## Discussion

This cohort study found higher rates of multiple threats and acts of violence early in the medical career (fourth year after graduation). Male physicians reported more multiple threats and multiple acts of violence in the 4th and 10th years after graduation, whereas there was no gender difference in the 15th and 20th years after graduation. With regard to personality, low levels of the vulnerability trait (neuroticism) in medical school were associated with more threats of violence later in the career. Working in the psychiatric department was an independently associated risk factor for both threats and acts of violence, as was being a male physician. We identified an association between cohort and WPV, indicating a reduction in WPV events in the younger cohort (medical student cohort).

To our knowledge, this is the first study to investigate the course of prevalence of WPV in individual physicians during the first 20 years of their medical career. Our analysis found that the prevalence of threats of violence decreased throughout this period. This is in line with a previous study among Saudi physicians that reported that younger respondents (aged <35 years) and those with fewer years of clinical experience (<10 years) reported a higher percentage of violent incidents.^19^ In contrast, a study of Norwegian physicians reported that there was more exposure to WPV with age, except for the oldest age group (aged >55 years).^8^ However, the latter was a cross-sectional study in which the responders were categorized into 3 age groups instead of 4 career stages as in our study. The higher prevalence of WPV in the fourth year after graduation may be due to several reasons. Physicians who recently graduated report high workload, stress, and a poor working environment.^36,37,38^ In turn, stress may lead to more patient conflicts and violence.^13^

We found that the 6-year older cohort experienced more patient violence than the younger cohort at the equivalent career stages. This is consistent with the study by Johansen et al,^8^ which identified a significant decrease in acts of violence experienced from 63.4% to 46.1% among psychiatrists between 1993 and 2014. There has been an increased focus on personnel security issues within psychiatric settings in Norway, which might be associated with this positive trend.^39,40^ Better risk assessment and more personalized and individual approaches have reduced violence in a ward with forensic psychiatric patients in Norway.^41^ In addition, there are lower levels of work stress in the younger NORDOC cohort,^21^ and this may have reduced levels of conflicts with their patients.^40^

The finding that male physicians report more acts and threats of violence concurs with findings among Norwegian physicians,^8^ as well as studies of physicians in New Zealand,^14^ and of police officers.^42,43^ A systematic review of WPV against health care workers reported no gender difference in threats nor acts of violence,^2^ probably because of the large differences in sample size between the 2 genders. Therefore, our finding of a significant gender difference in a longitudinal gender-balanced study among physicians is quite important. It is not clear whether men are attacked more because of the patients’ mere perception of a male physician, or because of characteristics in men’s behavior. A Norwegian study showed that a higher female proportion in psychiatry staff reduced rates of violence.^41^ One possible explanation is that the appearance of a male worker creates a higher level of threat to a male patient, with a higher expectance of physical aggression (intermale aggression).^44^ Research in psychology repeatedly shows gender differences in traits we have not controlled for in this study. For instance, women tend to be more agreeable than men, a trait consisting of 2 facets; politeness and compassion.^45^ To what degree such personality plays a part in this matter is highly uncertain.

An intriguing finding of this study was that high levels of vulnerability (or neuroticism) personality trait were associated with reduced risk of experiencing threats of violence. Interestingly, another study found that police officers with high vulnerability experienced stressful situations less frequently.^46^ Physicians who score high on vulnerability may avoid situations that can lead to WPV, and they may have better communication skills.^47^ We did not find vulnerability to be associated with acts of violence, but this may be a false-negative finding because of the low number of participants who experienced repeated acts of violence.

In terms of prevention, both the medical curriculum and postgraduate training should teach about the independent higher risks of being early in the career, male, and low in vulnerability trait. When meeting patients with a history of violence, caution and concern are key, and a physician with a low-vulnerability personality may be too little on alert and even too hazardous in his/her behavior. In addition, training of medical students and young physicians in handling and communicating with demanding, angry, and violence-simulated patients could be effective.^48^ Courses in self-defense strategies for the youngest physicians of both genders in venues at risk (eg, psychiatric and emergency department wards) could increase their self-efficiency in dealing with WPV, and even calm their patients. Future research should investigate the long-term effect of WPV on work impairment and sickness absence, particularly among female physicians,^49^ but also the preventive effect of targeting identified risk factors. In societies with higher rates of criminal violence, more structural and community-based preventive efforts may be necessary.^7^

### Strengths and Limitations

The major strengths of this study were the long-term longitudinal design throughout 20 years of the medical career and the investigation of multiple acts or threats of WPV. Furthermore, personality traits are seldom included in similar epidemiology surveys. The implementation of the vulnerability trait, which resembles negative affectivity, also adjusts the other self-reported findings for a negative affect response bias (eg, not only the most vulnerable individuals report more distress).^50^ We believe that the individual risk factors found in this study may be generalized to physicians in many other countries. The work-related factors may differ in other health care systems.

This study also had some limitations. One limitation was the loss of follow-up, which may have impacted the prevalence data; however, loss to follow-up would have less effect on the GEE analysis of factors related to WPV because this analysis is relatively robust to missing responses. The external validation may also be limited, except for other Northern European countries with sociodemocratic policies and a public health system. A major limitation is possible recall bias because measures were self-reported and there may be individual disparities in definition of threats and acts of violence. In addition, the first 2 WPV observations refer to different timespans than the last 2 observations. At T2, physicians had been working for only 4 years after leaving medical school, and we believe the WPV question captures about the same timespan (4-5 years) as T4 and T5. Therefore, we believe there was a true decline in WPV throughout the whole observation period. Nevertheless, the formulation “have you ever experienced” at T3 (10 years after graduation) may diverge from the other 3 and be less reliable for comparison. The immigration of a large number of physicians and the fact that many Norwegians have studied abroad during the last 20 years may mean that the NORDOC sample is less representative of Norwegian physicians in 2019.

## Conclusions

This cohort study found that being a male physician, having low levels of the vulnerability trait, being at an early career stage, and working in psychiatry were all independent risk factors associated with experiencing WPV. These findings suggest that preventive efforts should include early-career and male physicians, with additional emphasis on personality.
